# A new genus and species for the first recorded cave-dwelling Cavernicola (Platyhelminthes) from South America

**DOI:** 10.3897/zookeys.442.8199

**Published:** 2014-09-23

**Authors:** Ana Maria Leal-Zanchet, Stella Teles de Souza, Rodrigo Lopes Ferreira

**Affiliations:** 1 Instituto de Pesquisas de Planárias and Programa de Pós-Graduação em Biologia, Universidade do Vale do Rio dos Sinos – UNISINOS, 93022-000 São Leopoldo, RS, Brazil; 2 Centro de Estudos em Biologia Subterrânea, Setor de Zoologia Geral, Departamento de Biologia, Universidade Federal de Lavras, Campus Universitário, Caixa Postal 3037, Lavras, Minas Gerais, Brazil

**Keywords:** Troglobitic species, subterranean diversity, freshwater flatworms, triclads

## Abstract

Species diversity of Brazilian cave fauna has been seriously underestimated. A karst area located in Felipe Guerra, northeastern Brazil, which is a hotspot of subterranean diversity in Brazil, has revealed more than 20 troglobitic species, most of them still undescribed. Based on recent samplings in this karst area, we document the occurrence of the suborder Cavernicola (Platyhelminthes) in South American hypogean environments for the first time and describe a new genus and species for this suborder. *Hausera* Leal-Zanchet & Souza, **gen. n.** has features concordant with those defined for the family Dimarcusidae. The new genus is characterized by two unique features, viz. an intestine extending dorsally to the brain and ovovitelline ducts located dorsally to the nerve cords, which is complemented by a combination of other characters. The type-specimens of *Hausera hauseri* Leal-Zanchet & Souza, **sp. n.** are typical stygobionts, unpigmented and eyeless, and they may constitute an oceanic relict as is the case of other stygobiotic invertebrates found in this karst area in northeastern Brazil.

## Introduction

Species diversity of Brazilian cave fauna has been seriously underestimated (Ferreira 2005). Although more than 11,000 caves have been documented in Brazil, this may represent only 10% of the potential number of caves in the country (Auler et al. 2001). Studies performed in unexplored areas have revealed dozens of new troglobitic taxa, some of which were only recently described (Pellegrini and Ferreira 2014, Azara and Ferreira 2013, Iniesta and Ferreira 2013). One of the undescribed species, sampled in a karst area in northeastern Brazil, is a freshwater flatworm hereby assigned to the suborder Cavernicola (Platyhelminthes: Tricladida).

The triclad infraorder Cavernicola Sluys, 1990 was proposed to encompass five species of the family Dimarcusidae Mitchell & Kawakatsu, 1972 and now constitutes one of the three triclad suborders (Sluys et al. 2009). This taxon includes four genera, viz. *Rhodax* Marcus, 1946, *Opisthobursa* Benazzi, 1972, *Balliania* Gourbalt, 1972 and *Novomitchellia* Özdikmen, 2010 (Sluys 1990, Sluys et al. 2009, Özdikmen 2010). As its name indicates, most species of Cavernicola have type-localities in speleological habitats; they were recorded in Mexico, Tahiti Island and East Malaysia, respectively (Kawakatsu and Mitchell 1984, Sluys 1990). *Rhodax evelinae* Marcus, 1946 is an exception because its type-locality is in surface water in São Paulo city, southeast Brazil (Marcus 1946). Asexual specimens of presumed *Rhodax* were also recorded in surface water from southern Brazil (Braccini and Leal-Zanchet 2013, Vara and Leal-Zanchet 2013), and they have been introduced in tanks of tropical fishes in Japan (Kawakatsu et al. 1985, 1995, Sluys et al. 2010). In addition, the occurrence of a morphospecies of presumed *Opisthobursa* was reported in a hypogean environment in Guatemala after a morphological analysis of immature specimens (Kawakatsu and Mitchell 1983). Thus, the diversity of the suborder Cavernicola in South-American subterranean habitats is largely unknown.

After erecting the taxon Cavernicola and since the description of *Novomitchellia sarakawana* (Kawakatsu & Chapman, 1983), no new species have been described for the family Dimarcusidae. In addition, the only known Cavernicola species from South America is *Rhodax evelinae*, without records from subterranean habitats. Based on recent samplings in the Felipe Guerra karst area, northeastern Brazil, we provide here the first documentation of the family in South American hypogean environments by describing a new genus and species for this family.

## Material and methods

**Figures 1–5.**
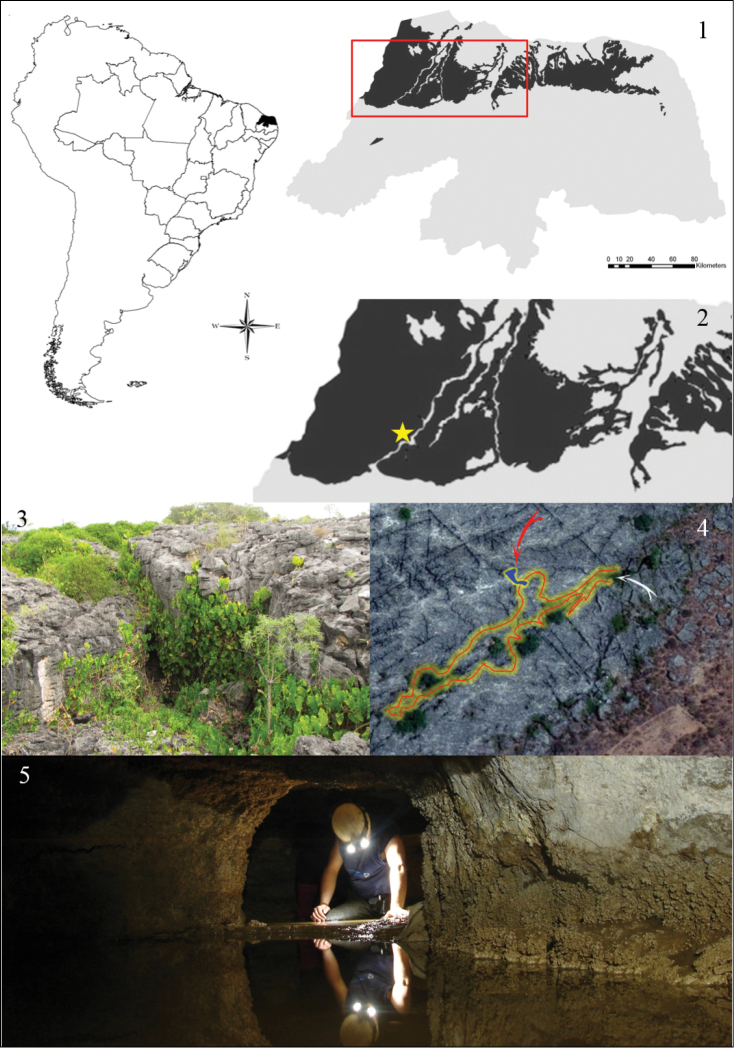
Type-locality of *Hausera hauseri* Leal-Zanchet & Souza, sp. n.: **1–2** location in Rio Grande do Norte, Brazil, showing the range of limestone outcrops from the Apodi Group in detail (black) and the location of the Crotes cave (star) **3–4** surroundings of the main cave entrance (**3**) and aerial view of the region (**4**) indicating the cave contours (red/yellow line), main entrance (white arrow) and secondary gallery (red arrow); **5** perennial stream representing the sampling site.

Specimens were collected from the Crotes cave, a limestone outcrop located in Felipe Guerra (5°33'38.87"S; 37°39'31.80"W), Rio Grande do Norte, Brazil (Figs 1–3). The type-locality is situated in the Caatinga biome, which is dominated by a semi-arid climate (type BSw’h’ of Köppen’s classification). This cave is part of a complex of 70 caves called “Lagedo do Rosário”, most of which have no perennial water bodies. The Crotes cave was formed by the expansion of a diaclasis, which is a diagenetic fracture in the rock. The main gallery of this cave has a 237 m linear projection, and the roof has several vertical openings. Despite the dominance of dry substrates, a small perennial stream (Fig. 4) drains water from the epicarstic system. The flatworms were directly sampled from this water body (Fig. 5), which has a water depth of 10−15 cm.

During the field work, the specimens were photographed and fixed in ethanol 70%. Fixed specimens were analysed and photographed with a stereomicroscope. They were dehydrated and embedded in Paraplast. This material was sectioned at 5−7 µm and stained with hematoxyline/eosine or Goldner’s Masson (Romeis 1989). Colour descriptors, based on the uptake of dyes of particular colours, were used for classifying secretions with trichrome methods: erythrophil, xanthophil and cyanophil. The term cyanophil is also applied to secretions that have an affinity for the green dye of Goldner’s Masson.

Type-material was deposited in the following reference collections: Museu de Zoologia da Universidade do Vale do Rio dos Sinos, São Leopoldo, Rio Grande do Sul, Brazil (MZU), and the Helminthological Collection of Museu de Zoologia da Universidade de São Paulo, São Paulo, São Paulo State, Brazil (MZUSP).

### Abbreviations used in figures

a: anterior tip; bc: bulbar cavity; br: brain; cg 1: cyanophil glands 1; cg 2: cyanophil glands 2; ci: cilia; cm: circular musculature; de: dorsal epidermis; eg: erythrophil glands; ej: ejaculatory duct; fgd: female genital duct; gic: genito-intestinal canal; i: intestine; in: insunk nuclei; lm: longitudinal musculature; m: mouth; ma: male atrium; o: ovary; ov: oviducts; pb: penis bulb; ph: pharynx; php: pharyngeal pouch; pp: penis papilla; r: rhabdites; rg: rhabditogen glands; s: sperm; sd: sperm duct; sg: shell glands; sv: seminal vesicle; t: testes; ve: ventral epidermis; vi: vitellaria; xg 1: xanthophil glands 1; xg 2: xanthophil glands 2.

## Systematic account

### Order Tricladida Lang, 1884
Suborder Cavernicola Sluys, 1990
Family Dimarcusidae Mitchell & Kawakatsu, 1972

#### Genus Hausera

Taxon classificationAnimaliaTricladidaDimarcusidae

Leal-Zanchet & Souza gen. n.

http://zoobank.org/9F362C51-4671-40B7-A28B-4DC46A59D0C5

##### Type-species.

*Hausera hauseri* Leal-Zanchet & Souza, sp. n. Monotypic

##### Diagnosis.

Dimarcusidae without eyes and without a copulatory bursa; female genital duct communicating with the intestine; ovovitelline ducts without caudal dichotomy, uniting to form a common ovovitelline duct; follicular testes; sperm ducts separately penetrating the penis bulb.

##### Distribution.

Felipe Guerra (Crotes cave), Brazil

##### Etymology.

The new genus is dedicated to the late Prof Dr Josef Hauser SJ as acknowledgement of his great enthusiasm for the study of freshwater flatworms. Gender: feminine.

##### Differentiation of the genus.

The specimens of *Hausera hauseri* show features concordant with the definition of the family Dimarcusidae, viz. common oviduct or diverticulum oriented perpendicular to the horizontal bursal canal or female genital duct, penis bulb provided with glandular elements, ovaries generally located at some distance posterior to the brain, vasa deferentia (= sperm ducts) uniting to extra-bulbar common vas deferens or penetrating separately the penis bulb and testicular follicles fused or discrete (Sluys 1990). The specimens herein described show cell bodies of the penis glands within the bulb, the horizontal orientation of the female genital duct combined with the dorsal opening of the common oviduct, sperm ducts penetrating separately the penis bulb and discrete testicular follicles. The ovaries are situated posterior to the brain, but are close to it.

It is worth mentioning that the specimens of *Hausera hauseri* have a connection with the intestine that could be confused with a copulatory bursa in which the branch of the intestine immediately posterior to the bursa may stain differently from other parts of the posterior intestinal branches. In all examined specimens, the connections with other parts of the posterior intestinal branches could be traced, leading to the conclusion that a bursa is absent in *Hausera hauseri*.

Similarly to *Rhodax*, *Hausera* gen. n. does not have a copulatory bursa. Other diagnostic characters of *Rhodax*, however, such as presence of eyes, fused testes, sperm ducts uniting before penetrating the penis bulb and ovovitelline ducts with a caudal dichotomy do not occur in *Hausera* gen. n. Similarly to *Opisthobursa*, the sperm ducts separately penetrate the penis bulb in the new genus, but the latter lacks a bursa, in contrast to the genus *Opisthobursa*.

#### Hausera hauseri

Taxon classificationAnimaliaTricladidaDimarcusidae

Leal-Zanchet & Souza sp. n.

http://zoobank.org/44FA7072-366B-4967-91D6-C81E7FFE43E8

##### Material examined.

**Holotype.** MZUSP PL. 1562: *coll*. R. Ferreira, 22 March 2011, Crotes cave, Felipe Guerra, RN, Brazil – sagittal sections on five slides.

**Paratypes.** Crotes cave, Felipe Guerra, RN, Brazil. MZU PL.00142: *coll*. R. Ferreira, 22 March 2011 – sagittal sections on 10 slides; MZU PL.00148: *coll*. R. Ferreira, 22 March 2011 – transverse sections on 9 slides; MZU PL.00149: *coll*. R. Ferreira, 05 June 2010 – anterior region: transverse sections on 12 slides, posterior region: sagittal sections on 20 slides.

##### Diagnosis.

*Hausera hauseri* can be distinguished from other species in the Dimarcusidae by (1) the numerous testicular follicles arranged in two irregular rows next to the margins of the body, extending from the level of the ovaries to the posterior end of the body; (2) the ovoid bulbar cavity with a posteriorly directed diverticulum into which the sperm ducts open; (3) a short and narrow transition region between the bulbar cavity and the ejaculatory duct; (4) the ovovitelline ducts arising from the lateral surface of the ovaries.

##### Description.

**Figures 6–8.**
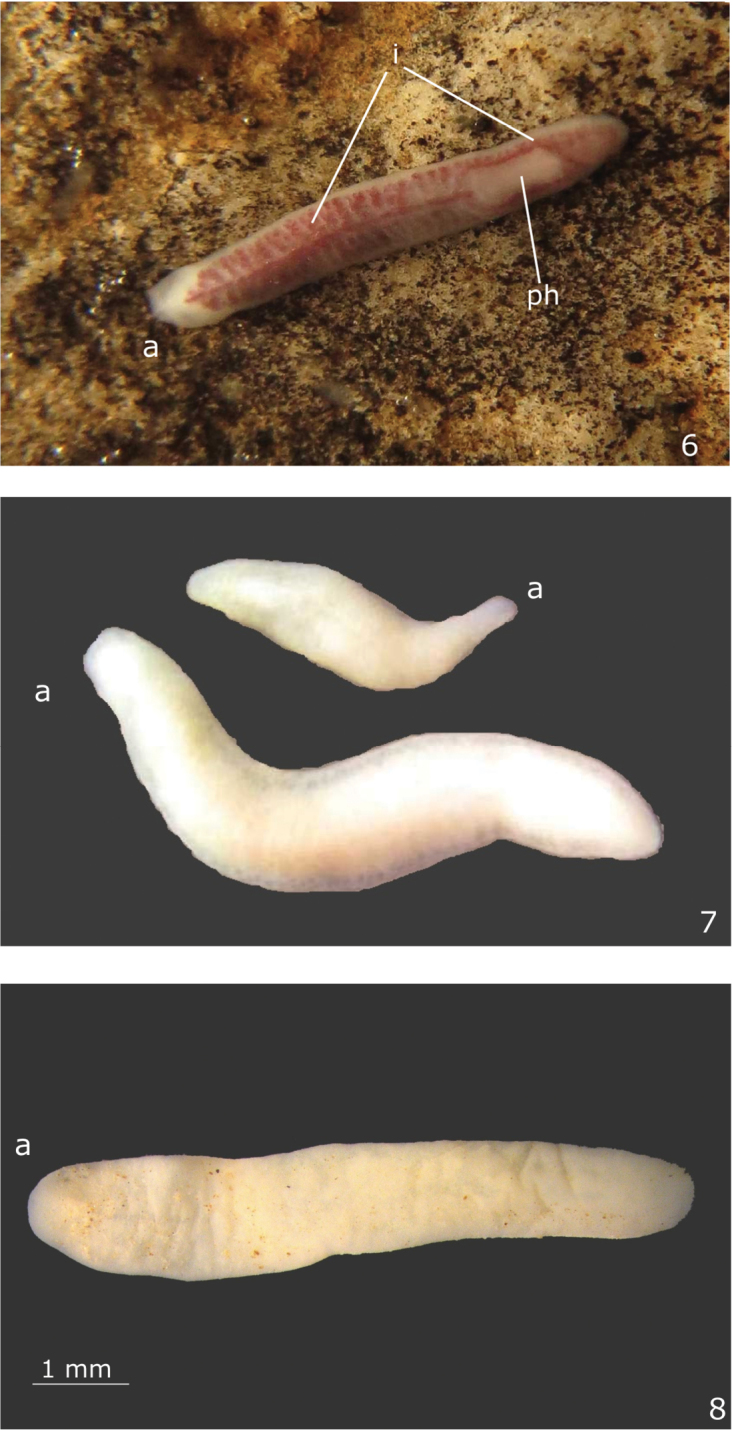
*Hausera hauseri* Leal-Zanchet & Souza, sp. n.: **6–7** photograph of live specimens in dorsal view, one of them (**6**) with intestine containing food and the pharynx visible **8** photograph of a preserved specimen (paratype MZU PL.00142) in dorsal view; grains of sand are seen over the dorsal surface. Scale bar for the figs **6–7** not available.

Live specimens unpigmented and eyeless (Fig. 6). Head truncate; posterior tip pointed (Figs 6–7). Preserved specimens up to 7.5 mm long and 2 mm wide (Table 1). Mouth and gonopore located at the posterior body third (Table 1). Body margins almost parallel (Figs 6–8). After fixation, anterior and posterior tips rounded (Fig. 8).

**Table 1.** Measurements, in mm, of specimens of *Hausera hauseri* Leal-Zanchet & Souza, sp. n. after fixation. DG: distance of gonopore from anterior end; DM: distance of mouth from anterior end. The numbers given in parentheses represent the position relative to body length. * Measurements done after histological processing.

	Holotype MZUSP PL. 1562	Paratype MZU PL.00142	Paratype MZU PL.00148	Paratype MZU PL.00149
Length	4.5	7	5	7.5
Width	1.5	2	2	1.5
Length*	4.2	6.7	4.7	7.2
DM*	2.7 (64%)	5 (75%)	2.6 (55%)	5.5 (76%)
DG*	3.6 (86%)	5.5 (82%)	3.4 (72%)	6 (83%)

**Figures 9–12.**
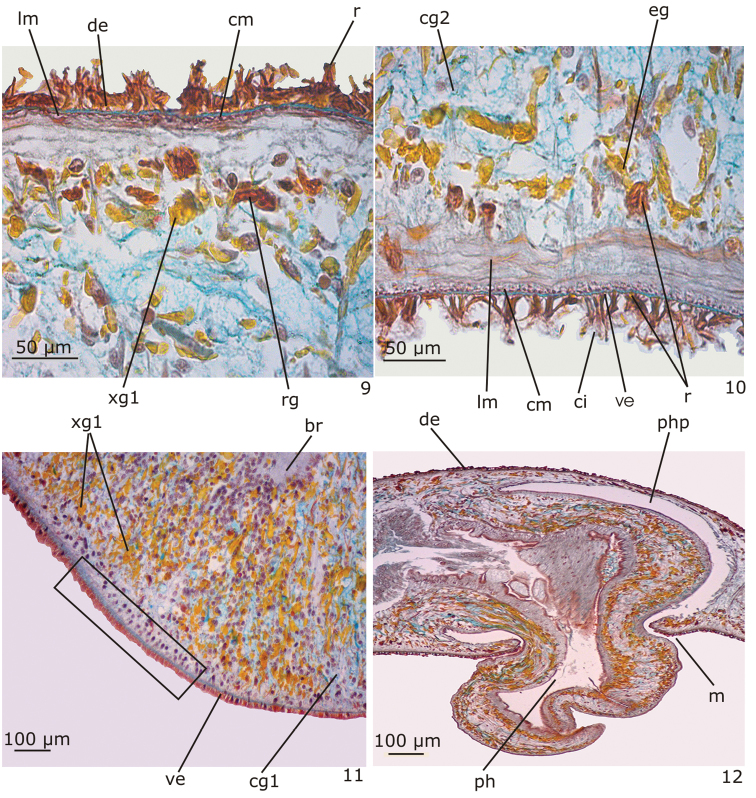
*Hausera hauseri* Leal-Zanchet & Souza, sp. n.: holotype (**9–10; 12**) and paratype MZU PL.00148 (**11**). **9–10** dorsal (**9**) and ventral (**10**) surfaces of the body in sagittal section **11** detail of the cephalic region in transverse section showing a sensory organ (rectangle) **12** sagittal section of the pharynx.

*Epidermis* (Figs 9–10). Columnar, ciliated epithelium, with abundant xanthophil, rhabidtogen secretion (rhammites), both dorsally and ventrally, being more densely distributed at the dorsal surface (Fig. 9). It also receives secretions of other four types of glands: (1) xanthophil, coarse granular secretion; (2) cyanophil amorphous secretion; (3) heavily stained cyanophil, fine granular secretion; (4) erythrophil, fine granular secretion (Figs 9–10). In addition, glands with heavily stained xanthophil, fine granular secretion open at the body margins and medially at the anterior and posterior tips of the body. Cilia more densely arranged on the ventral body surface (Fig. 10).

*Cutaneous musculature* (Figs 9–10). Two layers, viz. a thin subepithelial circular layer, followed by a thicker layer of longitudinal muscle. Dorsal and ventral cutaneous musculatures show similar height in the pre-pharyngeal region (13–15 µm thick). In the anterior region of the body, the ventral musculature (35 µm thick) is thicker than the dorsal one (9 µm thick).

*Sensory organs* (Fig. 11). Head with a pair of lateral sensory organs beginning about 140 µm after the anterior tip. They are lined with densely ciliated columnar epithelium, highly innervated, with insunk nuclei and receive few openings of secretory cells. The cutaneous musculature is very thin at the level of the sensory organs.

*Pharynx* (Fig. 12). Cylindrical; 0.9 mm long; it is located in the posterior third of the body. Pharynx and pharyngeal lumen lined with cuboidal ciliated epithelium with insunk nuclei. Three types of pharyngeal glands, viz. cells with coarse granular xanthophil secretion, cells with cyanophil amorphous secretion and less numerous cells with fine granular erythrophil secretion. Cell bodies of the pharyngeal glands located in the mesenchyme, mainly anterior and laterally to the pharynx. Outer musculature of the pharynx constituted of a thin subepithelial layer of longitudinal muscle, followed by a thin layer of circular muscle, each about 5 µm thick. Inner pharyngeal musculature composed of a thick subepithelial layer of circular fibres (about 25–28 µm thick), followed by a layer of longitudinal fibres (about 10 µm thick).

*Intestine* (Figs 19, 22, 23). Anterior intestinal trunk extending onto the posterior part of the brain (Figs 19, 23). Posterior intestinal trunks anastomose and communicate with the reproductive system through a genito-intestinal duct (Fig. 22). The part of the intestine that meets the genito-intestinal duct is lined with a tall columnar epithelium composed of cells with cyanophil cytoplasm and cells with coarse granular, erythrophil secretion; its lumen contains sperm and a small amount of erythrophil secretions (Fig. 22). Some spermatozoids are absorbed by the intestinal epithelium.

**Figures 13–17.**
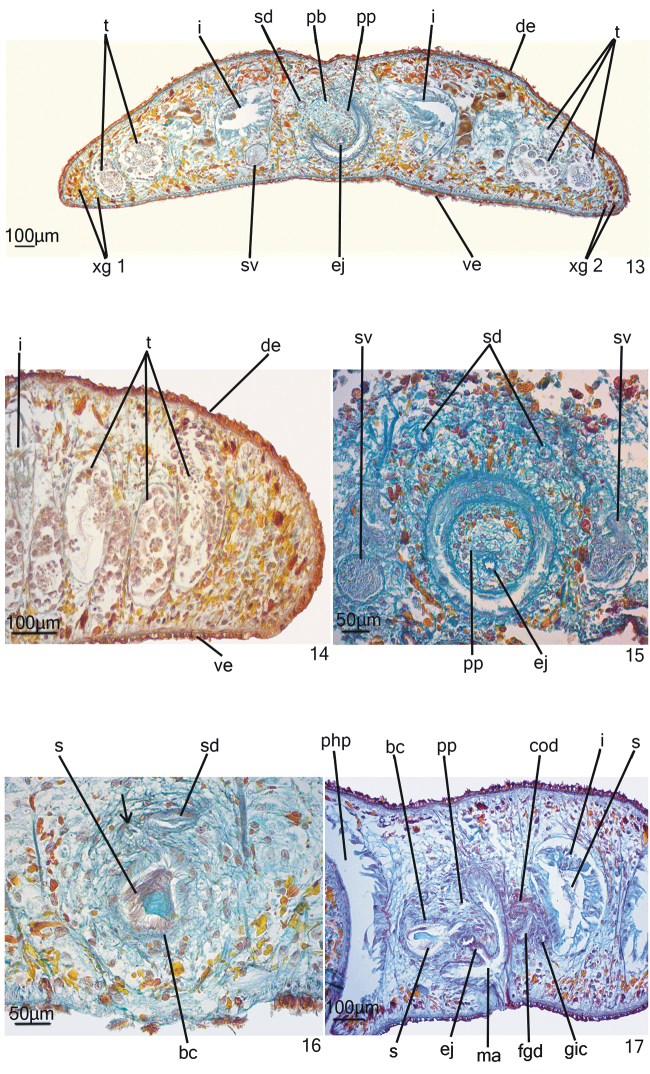
*Hausera hauseri* Leal-Zanchet & Souza, sp. n.: paratype MZU PL.00148 (**13; 15–16**) and holotype (**14; 17**) **13** transverse section of the body **14** sagittal section of the posterior tip **15–17** copulatory apparatus in transverse section (**15–16**) and sagittal section (**17**). The arrow indicates the diverticulum of the bulbar cavity.

**Figure 18.**
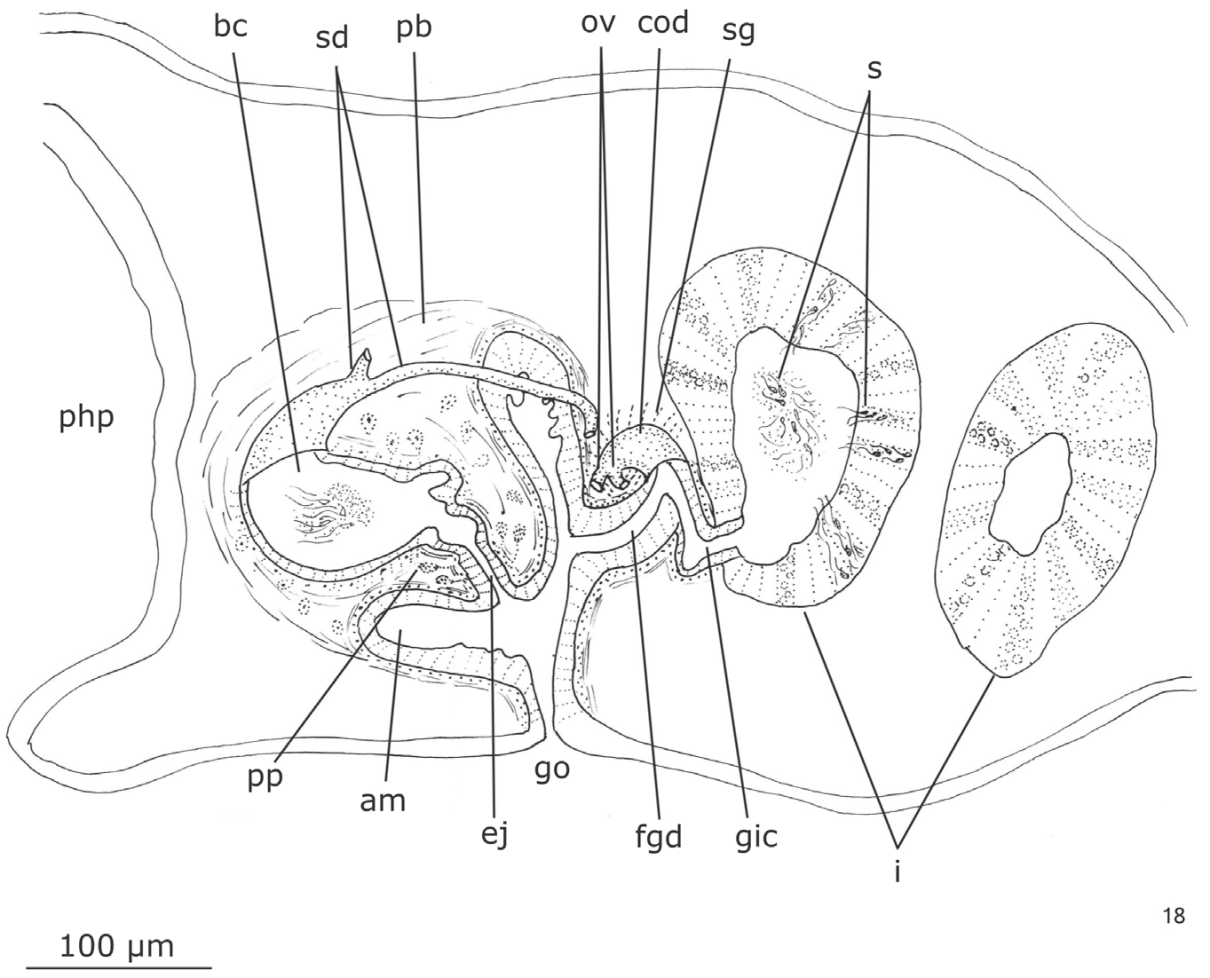
*Hausera hauseri* Leal-Zanchet & Souza, sp. n., holotype: sagittal composite reconstruction of the copulatory apparatus.

*Male reproductive system* (Figs 13–18). Numerous testicular follicles, 60–70 µm in diameter, arranged in approximately two irregular rows laterally to the intestinal diverticles, near body margins (Fig. 13). Testes extend from the level of the ovaries to the posterior end of the body (Figs 13–14). Sperm ducts form spermiducal vesicles laterally to the pharynx, diminishing in diameter laterally to the male copulatory apparatus (Fig. 15). They ascend, forming a loop, and then turn anteriad. Sperm ducts separately penetrate the penis bulb (Fig. 15) and open into the posteriorly directed diverticulum of the large bulbar cavity, in close proximity to each other (Fig. 16). Bulbar cavity single, ovoid, communicating with the ejaculatory duct through a short and narrow transition section (Figs 17–18). Ejaculatory duct opening at the tip of the conical and symmetrical penis papilla. The latter occupies almost the entire male atrium (Figs 13–15, 17–18).

Sperm ducts lined with a ciliated, cuboidal epithelium, underlain by a circular muscle layer. The large penis bulb consists of a loose connective tissue with weak interwoven muscle fibres (Figs 13–15, 17). Bulbar cavity lined with a ciliated, columnar epithelium, and coated with a subepithelial circular muscle layer, followed by a longitudinal muscle layer. Numerous glands with intrabulbar cell bodies and amorphous, cyanophil secretion open into the bulbar cavity. The bulbar cavity contains sperm and cyanophil secretion (Fig. 16). The transition part between the bulbar cavity and the ejaculatory duct is lined with a non-ciliated, cuboidal epithelium, the cells of which show an irregular height. A layer of circular muscle fibres surrounds the epithelial lining of the transition section. Ejaculatory duct lined with non-ciliated, cuboidal epithelium with irregular height, and surrounded by a subepithelial layer of circular muscle, followed by a layer of longitudinal muscle. This duct receives a xanthophil, fine granular secretion from intrapapillar glands. Penis papilla lined with a ciliated, columnar epithelium that becomes flat towards the tip of the papilla. Muscularis of penis papilla composed of a subepithelial layer of circular fibres and a layer of longitudinal fibres. Glands with amorphous, cyanophil secretion and with amorphous, xanthophil secretion open through the epithelial lining of the penis papilla. Both glands show intrapapillar cell bodies. Male atrium lined with a ciliated, high columnar epithelium, the cells of which have an irregular height (Fig. 17). The male atrial muscularis is constituted of a subepithelial layer of circular muscle, followed by a layer of longitudinal muscle. Glands with fine granular, cyanophil secretion and less numerous xanthophil glands with amorphous secretion open into the male atrium.

**Figures 19–24.**
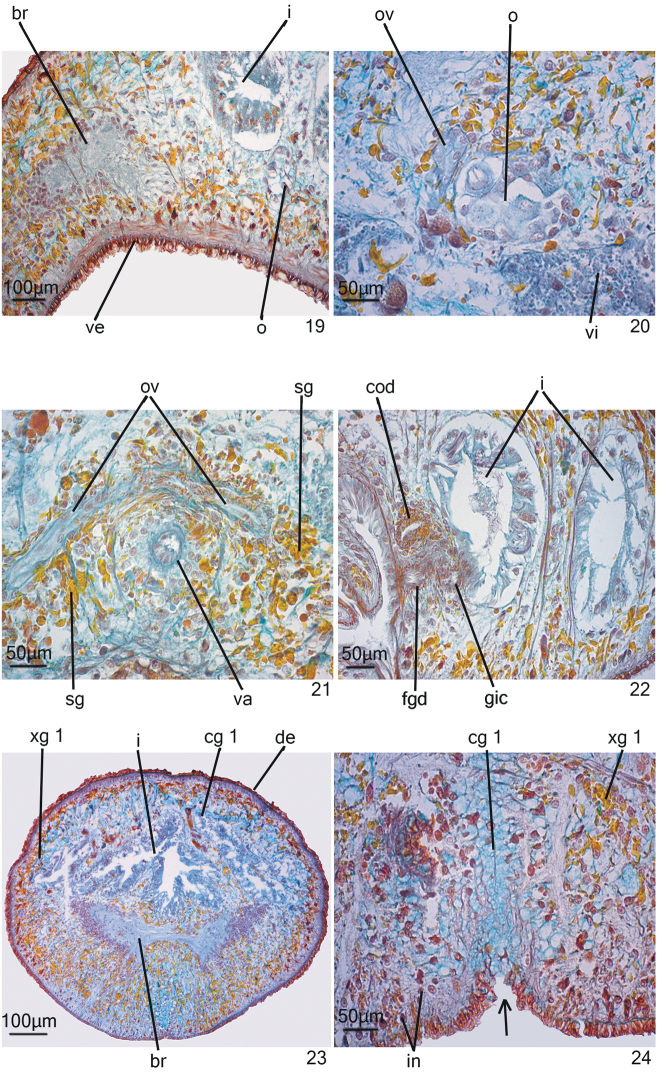
*Hausera hauseri* Leal-Zanchet & Souza, sp. n.: holotype (**19; 22**) and paratype MZU PL.00148 (**20–21; 23–24**). **19** cephalic region in sagittal section **20** detail of the ovary and ovovitelline duct in transverse section **21–22** detail of the copulatory apparatus in transverse section (**21**) and sagittal section (**22**) **23–24** cephalic region in transverse section. The arrow indicates a slit in the ventral surface of the body.

*Female reproductive system* (Figs 18, 19–22). Vitellaria poorly developed, located between intestinal branches. Ovaries, 70 µm – 100 µm in diameter, situated dorso-medially to the nerve cords, at about 0.6 mm from the anterior tip and close to the brain (110 µm posteriorly to the brain) (Fig. 19). Ovovitelline ducts arising from the lateral surface of the ovaries (Fig. 20) and running backwards dorsally to the nerve cords. At about the level of the gonopore, the ovovitelline ducts turn dorso-medially and form a common ovovitelline duct that is dorso-anteriorly directed (Figs 18, 21). Common ovovitelline duct opening into a female genital duct. The latter communicates with the male atrium and through a genito-intestinal canal with the intestine. The genito-intestinal canal is ventro-posteriorly directed (Figs 18, 22).

Ovovitelline ducts lined with ciliated, cuboidal epithelium, receiving the secretion of shell glands in their most posterior sections (Figs 18, 21). Common ovovitelline duct lined with ciliated, cuboidal or columnar epithelium, underlain by a circular muscle layer; it receives the secretion of shell glands (Fig. 18). These glands have a xanthophil, fine granular secretion (Fig. 21). Female genital duct and genito-intestinal canal lined with ciliated, cuboidal epithelium, receiving an amorphous, cyanophil secretion. The muscularis of the female genital duct and the genito-intestinal canal consists of a layer of circular fibres.

##### Etymology.

The new species is dedicated to the late Prof Dr Josef Hauser SJ.

##### Geographical distribution.

Known only from the type-locality, Felipe Guerra (Crotes cave), Brazil.

##### Variability.

Vitellaria well-developed in paratype MZU PL.00148 and poorly developed in paratypes MZU PL.00142 and PL.00149. In paratype MZU PL.00148, the anterior region of the body is subcylindrical in transverse section (Fig. 23) and shows a ventral median slit, posteriorly to the brain, where abundant cyanophil secretion is discharged (Fig. 24). In this paratype, the anterior intestinal branch extends slightly anterior to the brain. A short branch of each ovovitelline duct may extend anterior to the ovaries at least in paratype MZU PL.00148.

## Discussion

There are no genera within the Dimarcusidae into which we could comfortably include the species herein described; thus, the new genus *Hausera* is here proposed. Besides the combination of characters discussed above, the new genus is characterized by two unique features within its family: intestinal branch extending dorsally to the brain and ovovitelline ducts located dorsally to the nerve cords. According to Sluys (1990), the absence of a precerebral intestinal branch is a primitive character of the Dimarcusidae. Within this family, a precerebral intestinal branch is found only in *Opisthobursa josefinae* (Sluys 1990). In specimens of *Hausera hauseri*, the intestine generally extends dorsally to the brain, while it extends a little anterior to the brain only in paratype MZU PL.00148. The ovovitelline ducts are located medially to the nerve cords in the genera *Rhodax*, *Opisthobursa* and *Novomitchellia*, whereas they are located dorso-laterally to these cords in *Balliania* (Sluys 1990). The situation in *Hausera* differs from all these genera, in that the ovovitelline ducts are exactly dorsal to the nerve cords.

The type-locality of *Hausera hauseri*, a karst area located in Felipe Guerra, is unique in comparison with other karst areas in Brazil. Most limestone formations in Brazil are located in inner portions of the country, which in the past must have prevented marine groups from colonizing these caves. In contrast, the Felipe Guerra karst is located near the sea, and its limestone outcrops are at low altitude, which has allowed different invertebrates to colonize the caves during sea level rises in the past. Accordingly, many stygobiotic species found there represents oceanic relicts. They have evolved from marine ancestors trapped in the caves after isolation by events of introgression and regression of the ocean in the area, probably during the Terciary Period. This is the case of stygobiotic amphipods (Ferreira et al. 2010, Fiser et al. 2013) and may apply also to *Hausera hauseri*.

## Supplementary Material

XML Treatment for Hausera

XML Treatment for Hausera hauseri
